# COVID-19-Induced Hepatic Injury: A Systematic Review and Meta-Analysis

**DOI:** 10.7759/cureus.10923

**Published:** 2020-10-13

**Authors:** Sara Abdulla, Azhar Hussain, Dua Azim, Enas H Abduallah, Hayam Elawamy, Sundus Nasim, Sohail Kumar, Hassan Naveed

**Affiliations:** 1 Biochemistry, University of Benghazi, Benghazi, LBY; 2 Healthcare Administration, Franklin University, Columbus, USA; 3 Medicine, Xavier University School of Medicine, Oranjestad, ABW; 4 Internal Medicine, Dow Medical College, Dr. Ruth K. M. Pfau Civil Hospital, Karachi, PAK; 5 Mathematics, University of Tobruk, Tobruk, LBY; 6 Medical Laboratory, College of Medical Technology, Benghazi, LBY; 7 Internal Medicine, St. Matthew’s University School of Medicine, Grand Cayman, CYM

**Keywords:** sars-cov-2, covid-19, liver injury, hepatic injury, lfts, liver injury biomarkers, systematic review and meta-analysis

## Abstract

Background

The current pandemic of the novel coronavirus disease (COVID-19) is a global health challenge. Pulmonary dysfunction is the main outcome of COVID‐19 infection. In critically ill patients, however, liver complications have also been reported. Thus, we conducted a systematic review and meta-analysis to draw generalized conclusions regarding impaired liver biochemistry and its potential relationship with COVID-19 disease severity.

Materials and Methods

We searched the PubMed, Scopus, and Web of Science databases for all the related literature published up to June 20, 2020. The data were analyzed using R statistical software. A random‐effects model was employed for pooling the data. The risk of bias and quality of included studies was assessed using the modified Newcastle-Ottawa Scale (NOS) for cohort studies.

Results

The present meta-analysis comprises 10 retrospective and two prospective studies (6,976 COVID-19 patients). The serum analysis revealed significantly higher levels of alanine aminotransferases and aspartate aminotransferases and significantly lower albumin levels. Moreover, insignificant increases in serum levels of total bilirubin were observed. Upon subgroup analysis of six studies (severe cases, n=131; non-severe cases, n=334) stratified on the basis of disease severity, we found that these abnormalities were relatively higher in severe cases of COVID-19 (albumin [weighted mean difference (WMD), 34.03 g/L; 95% CI, 27.42 to 40.63; p<0.0001; I^2^=96.83%); alanine transaminase (ALT) [WMD, 31.66 U/L; 95% CI, 25.07 to 38.25; p<0.0001; I^2^=55.64%]; aspartate aminotransferase (AST) [WMD, 41.79 U/L; 95% CI, 32.85 to 50.72; p<0.0001; I^2^=51.43%]; total bilirubin [WMD, 9.97 μmol/L; 95% CI, 8.46 to 11.48; p<0.0001; I^2^=98%]) than in non-severe cases.

Conclusion

Deranged liver enzymes serve as prognostic factors to assess the severity of COVID-19. Liver markers should, therefore, be observed and monitored continuously.

## Introduction

In early December 2019, the novel coronavirus disease 2019 (COVID-19) was first identified in Wuhan City, China, as a cluster of rare cases of pneumonia [1]. Since then, the highly contagious COVID-19, caused by severe acute respiratory syndrome coronavirus 2 (SARS-CoV-2), has spread globally, causing substantial morbidity and mortality. With approximately 118,000 cases and 4,291 deaths recorded worldwide, this disease was declared a pandemic by the World Health Organization (WHO) on March 11, 2020. By June 30, 2020, a total of 10,185,374 confirmed cases and 503,862 deaths were documented in 216 countries [2].

The typical presentation of COVID-19 involves fever, weakness, nausea, and symptoms of pulmonary distress such as dry cough and dyspnea. However, the understanding and knowledge of the disease have improved over time, and it has become evident that SAR-CoV-2 damages not only the respiratory system but also the cardiovascular, gastrointestinal, and hepatobiliary systems, subsequently resulting in multi-organ failure (MOF) and death [3-4].

The involvement of the liver was also seen in the Middle East respiratory syndrome coronavirus (MERS-CoV) and SARS-CoV. Owing to their remarkable genetic similarity with SARS-CoV-2, liver involvement in COVID-19 was already predicted [5]. While the exact cause of hepatic injury is uncertain, the following major mechanisms have been suggested: (i) direct injury to hepatocytes or biliary epithelium; (ii) drug-induced hepatoxicity; (iii) liver injury related to exaggerated defense response of the body; and (iv) exacerbation of hepatic dysfunction by COVID-19 in individuals suffering from pre-existing liver disorders [6].

To date, however, literature regarding the correlation of COVID-19 with liver dysfunction has been minimal. Given the limited data, we aim to perform a meta-analysis and systematically review the current data available on liver injury in COVID-19 with two main objectives: (i) to draw more generalized conclusions about the abnormal serum markers of liver injury such as albumin, alanine aminotransferase (ALT), aspartate aminotransferase (AST), and bilirubin in laboratory-confirmed COVID-19 patients; and (ii) to determine its relationship with the severity of COVID-19.

## Materials and methods

Literature search strategy

We conducted this meta-analysis as per the guidelines provided by Preferred Reporting Items for Systematic Reviews and Meta-Analyses (PRISMA). The authors independently searched the Medline (PubMed interface), Scopus, and Web of Science databases using the keywords “COVID-19” or “2019-nCoV” and “laboratory data of Coronavirus infection” for all related publications up till June 20, 2020. The bibliography of relevant articles was scanned for any missed qualified paper. The electronic search strategy for all three databases is detailed in Table 1.

**Table 1:** Search strategy of electronic databases

Electronic Database	Search Strategy
PubMed	Search "COVID-19" Filters: Abstract; Humans
	Search “2019-nCoV” Filters: Abstract; Humans
	Search “laboratory data of Coronavirus infection “Filters: Abstract; Humans
	Search ((("COVID-19" AND has abstract[text] AND Humans[Mesh])) OR (“2019-nCoV” AND Has abstract[text] AND Humans[Mesh])) OR (“laboratory data of Coronavirus infection “ AND has abstract[text] AND Humans[Mesh]) Filters: Abstract; Humans
Scopus	Search "COVID-19" Filters: Abstract; Humans
Web of Science	Search "COVID-19" Filters: Abstract; Humans

Criteria for liver injury and disease severity

The present analysis involved adults with COVID-19 and associated liver damage, irrespective of their pre-existing chronic liver disease or COVID-19 severity. We described the liver injury as having serum alanine transaminase (ALT) and aspartate aminotransferase (AST) level >50 U/L and >40 U/L, respectively. Hypoalbuminemia was identified as serum albumin level <40 g/L; total bilirubin level >21 mmol/L was regarded as hyperbilirubinemia. Additionally, severity was defined according to the need for intensive care unit (ICU) admission, need for oxygen support, or death, or in parallel to the criteria explained in the studies.

Study selection

All extracted articles were tested for their eligibility based on the following inclusion criteria: (i) studies reporting reverse transcription-polymerase chain reaction (RT-PCR)-confirmed COVID-19 cases; (ii) reported studies of liver biomarkers (albumin, bilirubin, ALT, AST) and their mean serum levels among severe and non-severe cases of COVID-19; (iii) studies mentioning most of the laboratory data quantitatively, not qualitatively; and (iv) studies containing the characteristics and demographic information of the patients along with the year, country, number of patients, age, and sex.

We excluded the articles based on the following criteria: (i) articles that did not have a full-text link; (ii) studies reporting COVID-19 patients without laboratory diagnosis; (iii) case reports, case series, or any study having a sample size of less than 10; (iv) studies that lack relevant information for any reason; (v) papers that did not include primary information such as reviews, consensus, and guidelines; and (vi) studies involving pregnant women and children.

Data extraction and quality assessment

Initially, titles and abstracts were analyzed for inclusion criteria; the full text was examined in cases where the abstract was inadequate to assess whether the study met the inclusion criteria. For all eligible articles, data such as first author, year of publication, location, number of patients, age, sex, and serum levels of liver biomarkers (albumin, AST, ALT, and bilirubin) were extracted and recorded. The Microsoft Excel database (Microsoft Corporation, Redmond, Washington) was used to record all available laboratory data. Inconsistencies between the researchers were discussed to reach consensus.

The modified Newcastle-Ottawa Scale (NOS) for cohort studies was used to evaluate the quality and risk of bias of eligible papers. Studies with a NOS score of ≥5, 3-4, <3 were regarded as high, medium, and low-quality publications, respectively. We used the GRADEpro software (McMaster University, 2020, Hamilton, Canada) to assess the quality of evidence and graded it as high, moderate, low, and very low.

Statistical analysis

R statistical software (version 3.6.1; R Foundation for Statistical Computing, Vienna, Austria) was used to conduct statistical analysis. Additionally, the meta-package was employed to measure the proportion of COVID-19 positive individuals with deranged liver function tests (LFTs). First, we unified all the units of variables; we then expressed classified variables as percentages and continuous variables as median and inter-quartile range (IQR). Studies’ heterogeneity was assessed using Higgin's I-square (I^2^) test; I^2^ values of 0-25%, 25-50%, 50-75%, and >75% were indicated as insignificant, low, moderate, and high heterogeneity, respectively. We used the random-effect model for calculating the pooled median with a 95% confidence interval (CI) if I^2^≥50%; the fixed-effect model was selected if I^2^<50%.

A meta-analysis of variations in serum levels of albumin, AST, ALT, and total bilirubin was not carried out, as the selected studies presented the median values of lactate dehydrogenase (LDH) only; the reference ranges also varied among the studies due to different detection methods. Thus, a meta-analysis was conducted separately for each group of non-severe and severe patients and then compared.

## Results

Study selection process

**Figure 1:**
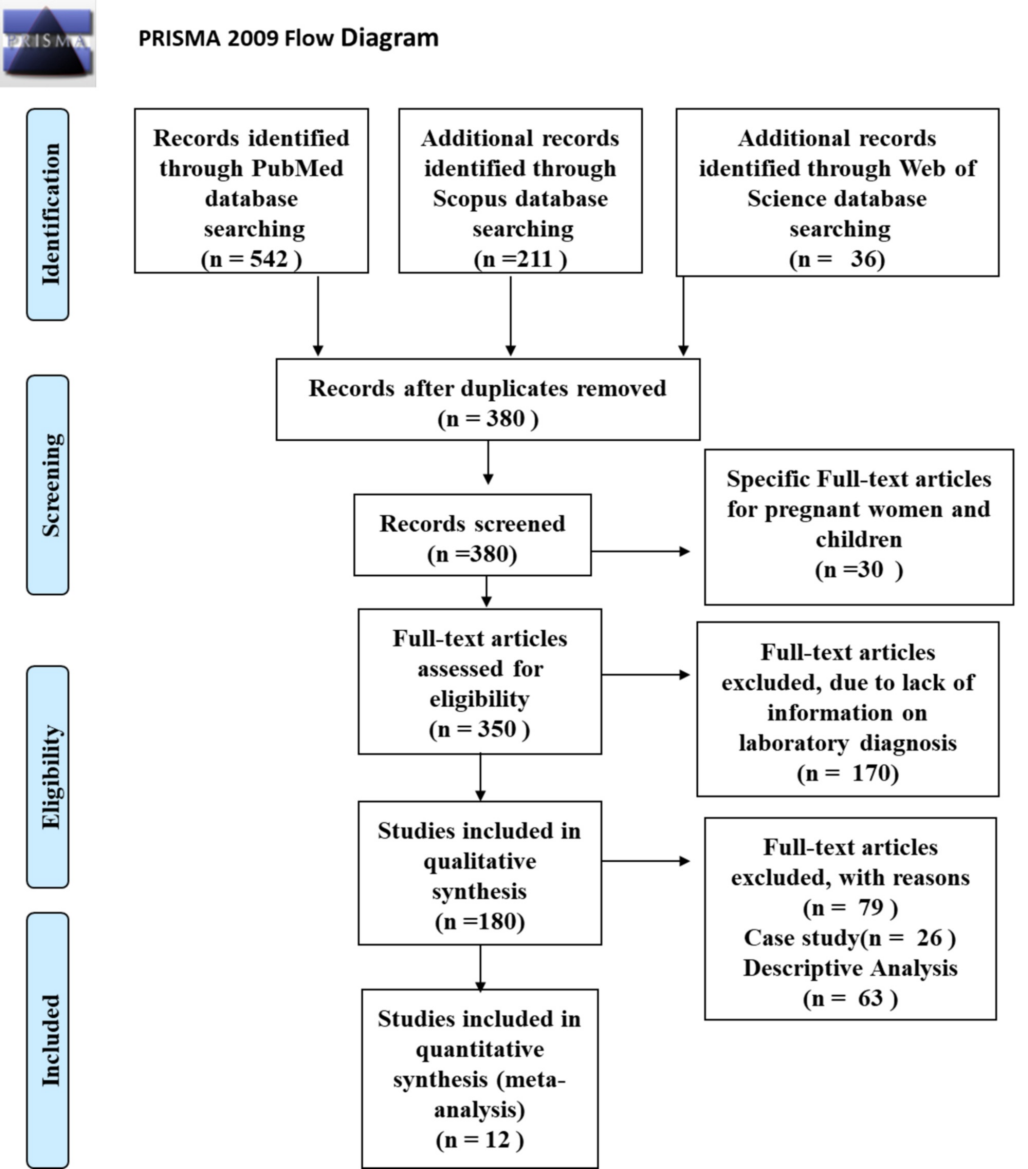
PRISMA flowchart showing the study selection process for the meta-analysis PRISMA: Preferred Reporting Items for Systemic Reviews and Meta-Analyses

The initial search yielded 789 articles. After removing 409 duplicates, a total of 380 articles were examined for title and abstract. Moreover, another 30 studies were excluded, as they specifically involved pregnant women and children. Thus, 350 papers were eligible for full-text screening. A total of 338 articles were excluded after reading the full text due to various reasons. Eventually, 12 studies, with a total number of 6,976 COVID-19 patients, were included in this systemic review and meta-analysis [1,3,7-16]. The study selection process using the PRISMA flow diagram is shown in Figure 1.

Study characteristics

Of the 12 studies, 10 had a retrospective [1,7-11,13-16] and two had a prospective study design [3,12]. Nine studies were from China while the remaining three were from the United States of America (USA), Italy, and Oman. Four studies reported multicenter data while the remaining eight studies reported single-center data. The sample size of studies varied between 21 and 5,700 patients with a mean age of 50.9 years (age range, 21-95 years). The results of the analysis demonstrated a male-dominant pattern; 60.2% of all the patients were male while the remaining 39.8% were females. All 12 studies were conducted in a hospital setting. The laboratory tests were obtained at the time of admission in each study included in the present meta-analysis. The characteristics of the studies enrolled in this analysis are listed in Table 2.

**Table 2:** Characteristics of the studies included in the meta-analysis CLD: chronic liver disease; NR: not reported ^a^Mean (range); ^b^Mean ± SD

Author	Year	Country	Study Design	Sample Size	Age, years	Female	Male	Baseline CLD	Follow-up Time
					Median (IQR)	n (%)	n (%)	(%)	(Days)
Wan S et al. [14]	2020	China	Retrospective	135	47 (36‐55)	63 (46.7)	72 (53.3)	1.48	16
Chen N et al. [1]	2020	China	Retrospective	99	55.5 (21-82)^a^	32 (32)	67 (68)	NR	20
Jin X et al. [10]	2020	China	Retrospective	74	46.14±14.19^b^	37 (50)	37 (50)	3.8	23
Feng Y et al. [8]	2020	China	Retrospective	476	53 (40-64)	205 (43)	271 (57)	NR	NR
Huang C et al. [3]	2020	China	Prospective	41	49·0 (41-58)	11 (27)	30 (73)	2.44	32
Richardson S et al. [13]	2020	USA	Retrospective	5700	63 (52-75)	2263 (39.7)	3437 (60.3)	0.52	35
Inciardi RM et al. [9]	2020	Italy	Retrospective	99	67±12^b^	19 (19.2)	80 (80.8)	NR	14
Wang D et al. [15]	2020	China	Retrospective	138	56 (42-68)	63 (45.7)	75 (54.3)	2.9	34
Khamis F et al. [11]	2020	Oman	Retrospective	63	48±16^b^	10 (15)	53 (85)	NR	61
Liu J et al. [12]	2020	China	Prospective	61	40 (1-86)	30 (49.2)	31 (50.8)	NR	10
Wang Z et al. [16]	2020	China	Retrospective	69	42 (35-62)	37 (54)	32 (46)	1.45	19
Chen G et al. [7]	2020	China	Retrospective	21	56 (50-65)	4 (19)	17 (81)	NR	NR

Laboratory data

Regarding the LFTs of COVID-19 positive patients, the most prevalent abnormality was reduced serum albumin level, whereas liver enzymes were mostly normal or marginally raised. Overall mean serum levels for albumin, ALT, AST, and total bilirubin are shown in Table 3. The blood biochemistry parameters for studies stratified based on the severity of COVID-19 are demonstrated in Table 4 [3,7,12,14-16].

**Table 3:** Liver function tests of the included studies ALT: alanine aminotransferase; AST: aspartate aminotransferase; NR: not reported

Author	Albumin (g/L; normal range, 40-55)	ALT (U/L; normal range, 9-50)	AST (U/L; normal range, 15-40)	Total Bilirubin (μmol/L; normal range, 0-21)
	Median (IQR)	Median (IQR)	Median (IQR)	Median (IQR)
Wan S et al. [14]	40.5 (37-43.4)	26 (12.9‐33.15)	33.4 (27.8‐43.7)	8.6 (5.9‐13.7)
Chen N et al. [1]	NR	39 (22-53)	34 (26-48)	15·1 (7·3)
Jin X et al. [10]	40.13 (35.95-42.6)	25 (15.75-38.47)	29.35 (20.87-38.62)	10 (7.15-13.8)
Feng Y et al. [8]	37.87 (32.8-41.84)	26 (16-41)	28 (21-39)	10.1 (7.5-14)
Huang C et al. [3]	31·4 (28·9-36)	32 (21-50)	34 (26-48)	11·7 (9·5-13·9)
Richardson S et al. [13]	NR	33 (21-55)	46 (31-71)	NR
Inciardi RM et al. [9]	33 (29.4-36)	34 (24-58)	46 (34-68)	NR
Wang D et al. [15]	NR	24 (16-40)	31 (24-51)	9.8 (8.4-14.1)
Khamis F et al. [11]	NR	NR	NR	10 (6-14)
Liu J et al. [12]	44 (50.5-47)	19 (14-33.5)	NR	NR
Wang Z et al. [16]	NR	23 (17-40)	28 (22-42)	NR
Chen G et al. [7]	33.7 (29.6-37.4)	26 (16-42)	27 (21-47)	8.8 (6.8-10.3)

**Table 4:** Liver function tests of the six studies stratified on the basis of severity ALT: alanine aminotransferase; AST: aspartate aminotransferase; NR: not reported

Authors	Severity of Disease	Albumin	ALT	AST	Total Bilirubin
		Median (IQR)	Median (IQR)	Median (IQR)	Median (IQR)
Wan S et al. [14]	Non-severe (n=95)	49.9 (37.4-43.6)	21.7 (14.8‐36.9)	22.4 (16.9-30.5)	8.6 (5.6‐14)
	Severe (n=40)	36.0 (33‐38.5)	26.6 (14.5‐33.3)	33.6 (25.7‐44.2)	9.8 (7.8‐15.6)
Huang C et al. [3]	Non-severe (n=28)	34·7 (30·2-36·5)	27.0 (19·5-40)	34.0 (24-40·5)	10·8 (9·4-12·3)
	Severe (n=13)	27·9 (26·3-30·9)	49.0 (29-115)	44.0 (30-70)	14.0 (11·9-32·9)
Chen G et al. [7]	Non-severe (n=10)	37.2 (35.8-38.8)	16.0 (13.3-21.8)	24.0 (21.5-26.5)	7.8 (6.4-9.5)
	Severe (n=11)	29.6 (28.6–33)	42.0 (32.5-50)	47.0 (28-74.5)	8.8 (7.9-10.5)
Liu J et al. [12]	Non-severe (n=44)	44.0 (41-47)	18.0 (14-32.3)	NR	NR
	Severe (n=17)	43.0 (37-45.5)	24.0 (14-34.5)	NR	NR
Wang Z et al. [16]	Non-severe (n=55)	NR	24 (16-40)	26 (21-39)	NR
	Severe (n=14)	NR	31.5 (23-52)	40.5 (24-62)	NR
Wang D et al. [15]	Non-severe (n=120)	NR	23.0 (15-36)	29.0 (21-38)	9.3 (8.2-12.8)
	Severe (n=36)	NR	35.0 (19-57)	52.0 (30-70)	11.5 (9.6-18.6)

Pooled analysis of laboratory findings

**Figure 2:**
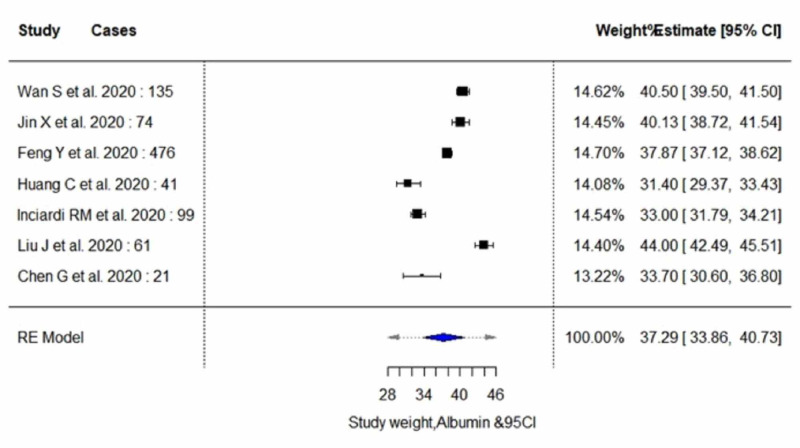
Forest plots for pooled analysis of serum levels of albumin using a random‐effects model CI: confidence interval

**Figure 3:**
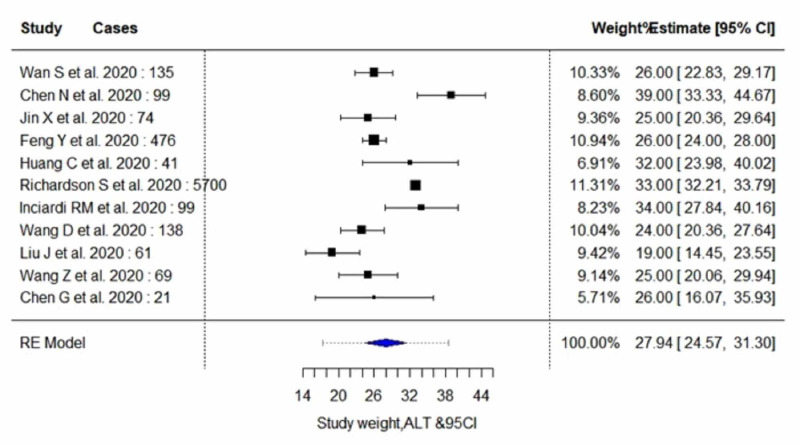
Forest plots for pooled analysis of serum levels of ALT using a random‐effects model CI: confidence interval; ALT: alanine aminotransferase

**Figure 4:**
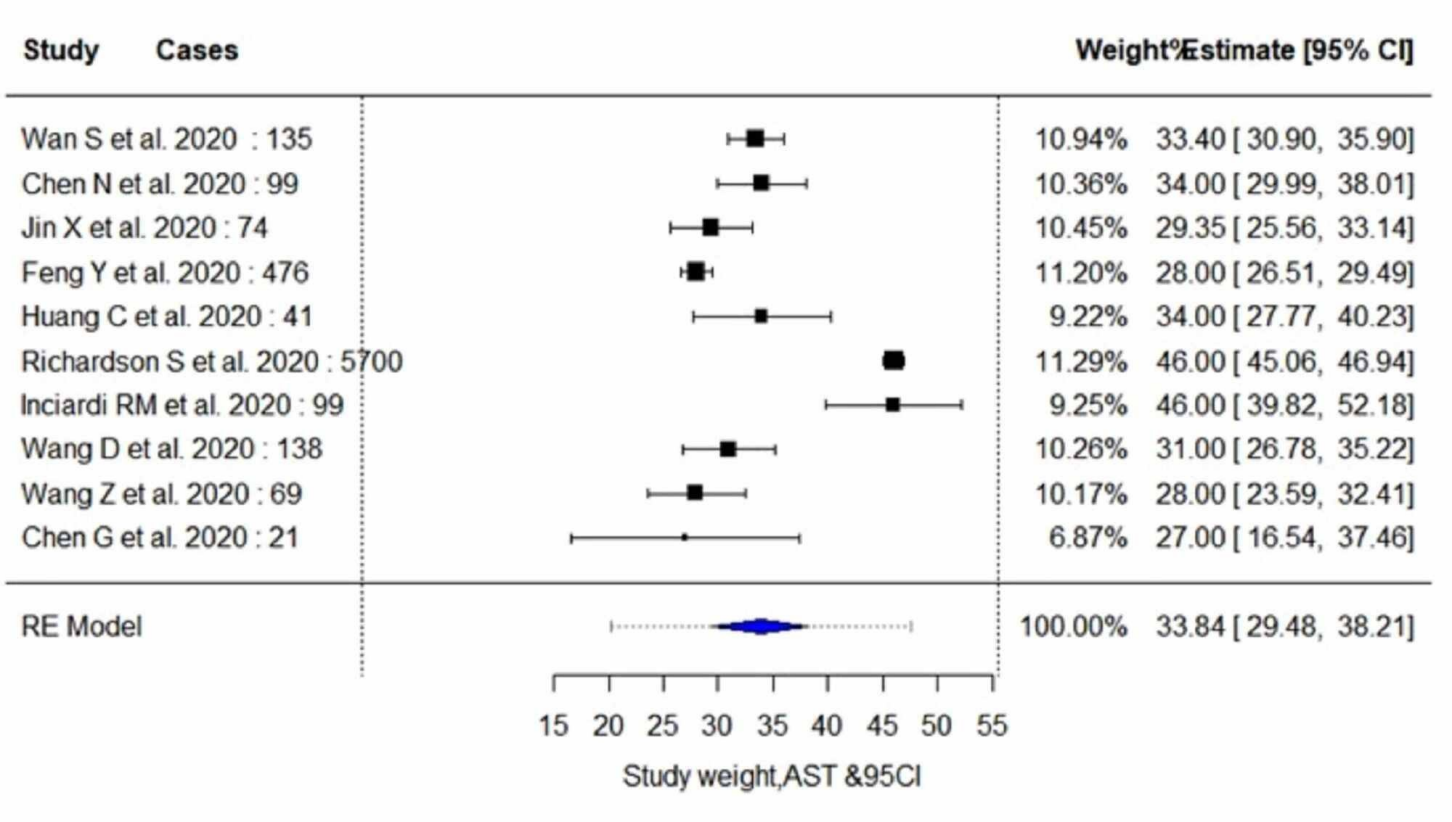
Forest plots for pooled analysis of serum levels of AST using a random‐effects model CI: confidence interval; AST: aspartate aminotransferase

**Figure 5:**
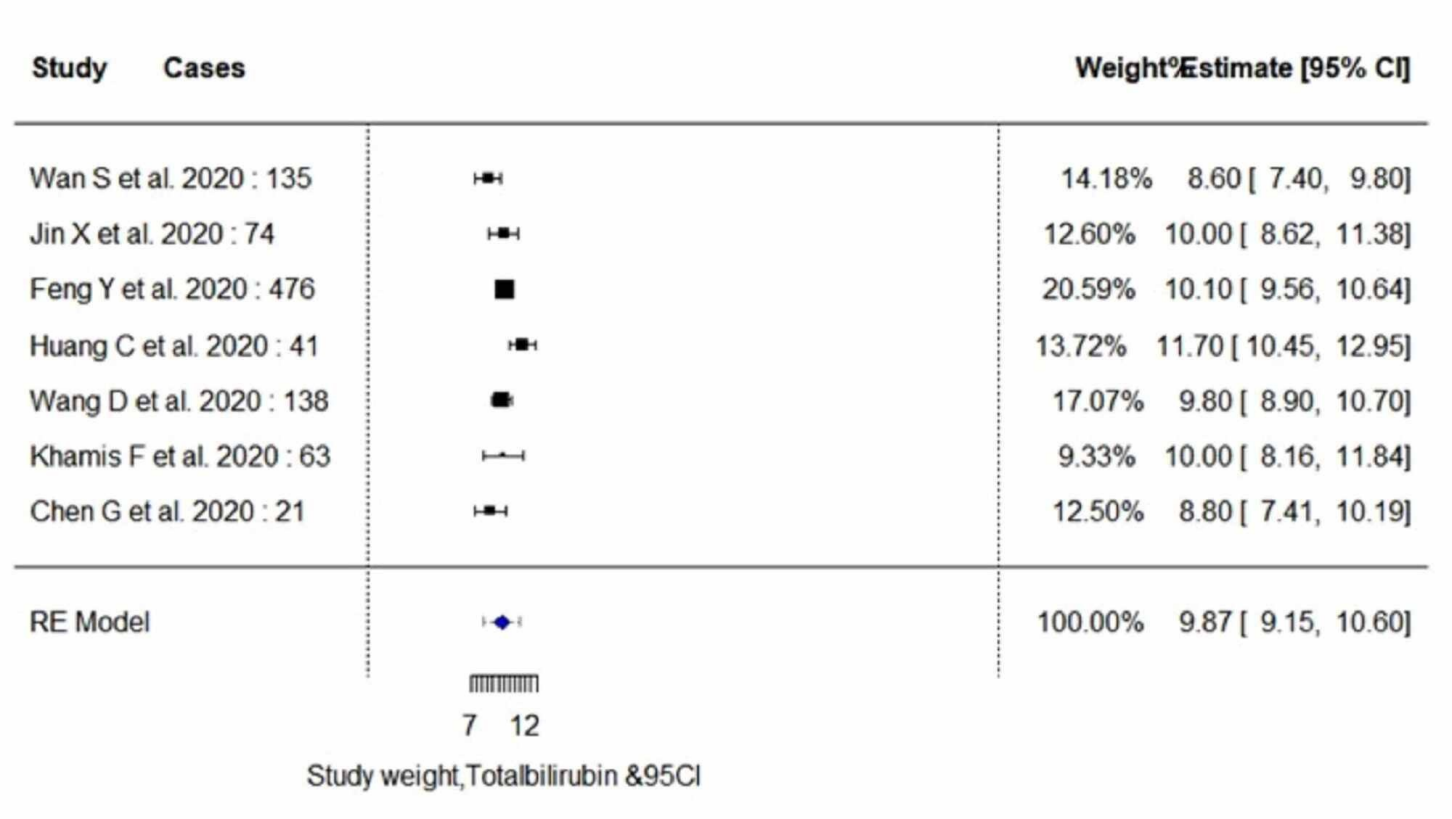
Forest plots for pooled analysis of serum levels of total bilirubin using a random‐effects model CI: confidence interval

The pooled proportion of abnormal LFT parameters in COVID-19 positive patients showed significantly lower serum levels of albumin (estimate, 37.29; 95% CI, 33.85 to 40.72; I^2^=97.99%; p_heterogeneity_<0.0001), and significantly higher serum levels of AST (estimate, 33.84; 95% CI, 29.47 to 38.20; I^2^=96.03%; p_heterogeneity_<0.0001) and ALT (estimate, 27.93; 95% CI, 24.57 to 31.30; I^2^=91.35%; p_heterogeneity_<0.0001). In addition, an insignificant increase in the serum levels of total bilirubin (estimate, 9.87; 95% CI, 9.14 to 10.59; I^2^=66.78%; p_heterogeneity_<0.0173) was observed. A random-effect method was adopted since the heterogeneity between the studies was high for all four parameters. Pooled estimates of LFTs are demonstrated in Table 5. The individual forest plots for pooled estimates of serum levels of albumin, ALT, AST, and total bilirubin are shown in Figure 2, Figure 3, Figure 4, and Figure 5, respectively. Moreover, upon quality assessment of the outcomes, they were rated to be of high quality. The quality of evidence is outlined in Table 5.

**Table 5:** Incidence of abnormal liver function tests: result of meta-analysis CI, confidence interval; ALT, alanine aminotransferase; AST, aspartate aminotransferase N^a^, number of studies; N^b^, number of patients

Variable	N^a^	Estimate	95% CI	N^b^	Standard Error	p-value	Measure of Heterogeneity				Quality of Evidence (GRADE)
							T^2^	Q	p	I^2^	
Albumin	7	37.29	33.85-40.72	907	1.752	<0.0001	20.730	207.597	<0.0001	97.99%	⨁⨁⨁⨁ HIGH
ALT	11	27.93	24.57-31.30	6913	1.716	<0.0001	25.894	120.37	<0.0001	91.35%	⨁⨁⨁⨁ HIGH
AST	10	33.84	29.47-38.20	6852	2.22	<0.0001	45.770	522.047	<0.0001	96.03%	⨁⨁⨁⨁ HIGH
Total Bilirubin	7	9.87	9.14-10.59	948	0.3698	<0.0001	0.589	15.406	0.0173	66.78%	⨁⨁⨁⨁ HIGH

Subgroup analysis

Six studies with 465 patients (severe cases, n=131; non-severe cases, n=334) were stratified based on the disease severity. Table 6 and Table 7 demonstrate the pooled estimates of LFTs of patients in the non-severe and severe group, respectively. 

**Table 6:** Pooled estimates of abnormal liver biochemical indicators of patients in non-severe group CI: confidence interval; ALT: alanine aminotransferase; AST: aspartate aminotransferase N^a^: number of studies; N^b^: number of patients

Variable	N^a^	Estimate	95% CI	N^b^	Standard Error	p-value	Measure of Heterogeneity			
							T^2^	Q	p	I^2^
Albumin	6	41.53	34.80-48.25	334	3.4312	<0.0001	45.98	182.65	<0.0001	98.33%
ALT	6	21.60	18.70-24.51	334	1.48	<0.0001	5.80	9.21	0.0669	51.54%
AST	6	26.66	22.93-30.38	334	1.89	<0.0001	14.38	20.44	0.0004	82.78%
Total Bilirubin	6	9.04	7.93-10.14	334	0.5618	<0.0001	1.10	14.74	0.0053	73.32%

**Table 7:** Pooled estimates of abnormal liver biochemical indicators of patients in severe group CI, confidence interval; ALT, alanine aminotransferase; AST, aspartate aminotransferase N^a^, number of studies; N^b^, number of patients

Variable	N^a^	Estimate	95% CI	N^b^	Standard Error	p-value	Measure of Heterogeneity			
							T^2^	Q	p	I^2^
Albumin	6	34.03	27.42-40.63	131	0.2922	<0.0001	43.66	66.11	<0.0001	96.83%
ALT	6	31.66	25.07-38.25	131	3.3641	<0.0001	33.52	10.74	0.0567	55.64%
AST	6	41.79	32.85-50.72	131	4.5584	<0.0001	48.61	8.65	0.0704	51.43%
Total Bilirubin	6	9.97	8.46-11.48	131	0.7715	<0.0001	0.81	4.81	<0.0001	98%

Serum Albumin

Of six studies included in the subgroup analysis, four studies with 258 subjects (severe cases, n=81; non-severe cases, n=177) reported outcome data on serum albumin levels, as demonstrated in Table 6 and Table 7 [3,7,12,14]. The random-effect result of these studies demonstrated that patients in the severe group had lower serum levels for albumin in comparison to the non-severe individuals (weighted mean difference [WMD], 34.03 g/L; 95% CI, 27.42 to 40.63; p<0.0001 vs WMD, 41.53 g/L; 95% CI, 34.80 to 48.25; p<0.0001). On analysis, both the subgroups yielded high heterogeneity (I^2^=96.83% vs 98.53%; p<0.0001).

Serum Alanine Aminotransferase (ALT)

All six studies with 468 subjects (severe cases, n=131; non-severe cases, n=334) included in the subgroup analysis reported outcome data on serum ALT levels, as demonstrated in Table 6 and Table 7 [3,7,12,14-16]. The random-effect result of these studies demonstrated that patients in the severe group had higher serum levels for ALT in comparison to the non-severe individuals (WMD, 31.66 U/L; 95% CI, 25.07 to 38.25; p<0.0001 vs WMD, 21.60 U/L; 95% CI, 18.70 to 24.51; p<0.0001). On analysis, both the subgroups yielded moderate heterogeneity (I^2^=55.64%; p=0.0567 vs I^2^=51.54%; p=0.0669).

Serum Aspartate Aminotransferase (AST)

Of six studies included in the subgroup analysis, five studies with 404 subjects (severe cases, n=114; non-severe cases, n=290) reported outcome data on serum AST levels, as demonstrated in Table 6 and Table 7 [3,7,14-16]. The random-effect result of these studies demonstrated that patients in the severe group had higher serum levels for AST in comparison to the non-severe individuals (WMD, 41.79 U/L; 95% CI, 32.85 to 50.72; p<0.0001 vs WMD, 26.66 U/L; 95% CI, 22.93 to 30.38; p<0.0001). On analysis, the severe group yielded moderate heterogeneity (I^2^=51.43%; p=0.0704), whereas the non-severe group yielded high heterogeneity (I^2^= 82.78%; p=0.0004).

Serum Total Bilirubin

Of six studies included in subgroup analysis, four studies with 335 subjects (severe cases, n=100; non-severe cases, n=235) reported outcome data on serum bilirubin levels, as demonstrated in Table 6 and Table 7 [3,7,14,15]. The random-effect result of these studies demonstrated that patients in the severe group had higher serum levels for total bilirubin in comparison to the non-severe individuals (WMD, 9.97 μmol/L; 95% CI, 8.46 to 11.48; p<0.0001 vs WMD, 9.04 μmol/L; 95% CI, 7.93 to 10.14; p<0.0001). On analysis, the severe group yielded high heterogeneity (I^2^=98%; p<0.0001), whereas the non-severe group yielded moderate heterogeneity (I^2^=73.32%; p=0.0053).

Risk of bias and quality assessment

Overall, six studies scored ≥5 and were, therefore, regarded as high-quality publications. Four studies scored between 3 and 4 and were considered to be of medium-quality; the remaining two studies scored <3 and were considered to be of low-quality, as demonstrated in Table 8.

**Table 8:** Summary of quality assessment and risk of bias using the modified Newcastle-Ottawa scale (NOS)

Criteria	Wan S et al. [14]	Chen N et al. [1]	Jin X et al. [10]	Feng Y et al. [8]	Huang C et al. [3]	Richardson S et al. [13]	Inciardi RM et al. [9]	Wang D et al. [15]	Khamis F et al. [11]	Liu J et al. [12]	Wang Z et al. [16]	Chen G et al. [7]
Representation of average adult in community (population-based=1 point; multicenter=0.5 point; single center=0 point)	0	0	0.5	0.5	0	0.5	0	0	0.5	0	0	0
Cohort size (>100 subjects=1 point; between 50-99 subjects=0.5 point; <50 subjects=0 point)	1	0.5	05	0.5	0	1	0.5	1	0.5	0.5	0.5	0
Reported information on percentages and pattern of liver injury (information with clarity=1 point; information derived from percentages=0.5 point; unclear=0 point)	1	1	1	1	1	1	0	1	0	0	0	1
Reported percentages of subjects with chronic liver disease at baseline (yes=1 point; no=0 point)	1	1	1	1	1	1	0	1	0	0	0	1
Assessed factors between mild and severe COVID-19 (yes=1 point; no=0 point)	1	0	0	0	1	0	0	1	0	1	0	1
Adequate clinical assessment (yes=1 point; no=0 point)	1	1	1	1	1	1	1	1	1	1	1	1
Sufficient follow-up period for outcome to occur (yes=1 point; unclear=0 point)	1	1	0	1	1	1	0	0	0	1	1	0
Adequate follow-up (all subjects were followed-up=1 point; >50% subjects were followed-up=0.5 points; <50%subjects were followed-up=0 point)	1	1	0	0	1	1	0	0	0	0	1	0
Total NOS Score	7	5.5	4	5	6	6.5	1.5	5	2	3.5	3.5	4

## Discussion

The present meta-analysis of 10 retrospective and two prospective studies investigated the possible link between impaired liver biochemistry and COVID-19 disease severity. Recent studies have shown that 37.2% to 76.3% of the patients infected with SARS-CoV-2 have impaired liver function [17-18].

In this meta-analysis, the laboratory findings revealed significantly lower levels of albumin and significantly higher levels of ALT and AST in COVID-19 patients; moreover, we also observed statistically insignificant higher levels of total bilirubin. Our results are in line with the previous researches on COVID-19, which also revealed hypoalbuminemia accompanied by elevated serum levels of aminotransferases and bilirubin as main indicators of liver injury [19-20]. The occurrence of hypoalbuminemia can be plausibly explained by the fact that albumin is a negative acute phase reactant, not a consequence of liver dysfunction.

The current suggests that critically ill COVID-19 positive individuals have a higher proportion of deranged liver biochemistries as compared to patients with a milder disease [21]. Upon comparing the LFTs of the severe and non-severe group, our results confirmed that patients with the severe clinical presentation of COVID-19 had lower levels of albumin and higher levels of total bilirubin, ALT, and AST relative to their counterparts. Recently, Guan et al. observed that approximately 28% and 56% of severely ill COVID-19 patients had increased serum levels of ALT and AST, respectively; only 20% and 18% of the patients with non-severe COVID-19 showed higher serum ALT and AST levels, respectively [22]. The elevations, however, cannot be unequivocally linked to direct viral assault on the liver. However, evidence indicates that aminotransferases are a surrogate indicator of chronic inflammation and increased oxidative stress, which offers a possible explanation for their elevation during a viral illness [23].

Hepatic damage has been recognized as a significant prognostic factor for poorer outcomes of SARS-CoV and MERS-CoV infections. COVID-19 also causes liver injury; however, the exact mechanisms of liver injury remain unclear [5]. Recently, hepatic postmortem biopsies performed in deceased COVID‐19 patients revealed moderate microvascular steatosis along with mild lobular and portal activity. The results were suggestive of either a direct effect of SARS‐CoV‐2 infection on the liver or drug-induced hepatoxicity [24].

Four possible explanations of COVID-19-induced hepatic damage have currently been proposed. The first proposed mechanism is a direct attack on hepatocytes or biliary epithelium by SARS-CoV-2, leading to deranged LFTs. Both the liver and bile duct cells express angiotensin-converting enzyme 2 (ACE2) receptors that are the binding site for cellular entry by SARS-CoV-2 [5-6]. Although the direct entry of the virus into the hepatocytes via the ACE2 receptor appears to be the most logical explanation of liver injury, evidence suggests that hepatocytes do not express high levels of ACE2 receptors, making the liver an improbable target for infection. Additionally, a preliminary study by Chai et al. revealed a high level of ACE2 expression in cholangiocytes, implying that COVID-19-associated hepatic dysfunction may occur from injury to bile duct cells; however, consistency in the elevation of alkaline phosphatase (ALP) in COVID-19 patients has not been observed, further providing evidence against the proposed mechanism [25].

The second proposal suggests treatment side effects as a possible cause of hepatotoxicity in SARS-CoV-2 infection. Medicines like acetaminophen, hydroxychloroquine, and other antivirals that are commonly used against SARS infections may cause liver damage [5-6]. However, there is little evidence that drug combinations currently available to treat COVID-19 infection compromise liver function. Fan et al. conducted a study on 148 patients with COVID-19-associated liver damage. The authors proposed that liver dysfunction may be due to the antivirals, lopinavir/ritonavir, used to treat SARS-CoV-2 infection [17]. Furthermore, results by Cai et al. also indicate that the hepatic damage seen in hospitalized patients with COVID-19 may be due to the adverse effects of drugs. They also found that patients with deranged LFTs were at a greater risk of severe disease progression [18].

The third mechanism suggested pertains to immune-mediated liver damage. There is a possibility that the hyperactivated defense system of the body in response to the virus may lead to the development of a cytokine storm and subsequent liver impairment [6,26]. Lastly, it has been speculated that SARS-CoV-2 may worsen already-compromised hepatic function in patients with pre-existing liver diseases such as viral hepatitis [5,26].

Here, it is also imperative to shed light on other causes of liver damage such as hepatic hypoxia due to pulmonary insufficiency, pulmonary embolism, low cardiac output, and cerebral respiration insufficiency. Hypoxic hepatitis, also referred to as shock liver and ischemic hepatitis is attributed to systemic hypotension or hypoxemia, resulting in decreased blood flow to the hepatocytes [27]. It is pre-established that patients with a severe presentation of COVID-19 develop pneumonia with subsequent progression to acute respiratory distress syndrome, septic shock, and ultimately death [3]. In addition, the occurrence of pulmonary embolism has been identified in some patients with SARS-COV-2 infection, resulting in acute respiratory insufficiency [27-28]. Thus, hepatic injury in COVID-19 could be plausibly explained by the fact that respiratory insufficiency (PaO2 <45 mmHg) could lead to liver injury due to reduced oxygen supply to the hepatocytes, which may eventually progress to liver failure.

Although SAR-COV-2 infection mainly attacks the respiratory system, invasion of the cardiovascular system by the virus is not uncommon. A retrospective study conducted in China among patients who died of COVID-19 revealed cardiac damage in 89% and cardiac failure in 14.6% of the patients [20]. The study also revealed that cardiac failure was the third most common cause of death after acute respiratory failure and sepsis syndrome/MOF [20]. Low cardiac output secondary to cardiac failure, as seen in respiratory disease and septic shock, may also predispose to liver injury and hypoxic hepatitis. The primary pathophysiology involves hepatic congestion from right heart failure along with reduced blood flow to the liver or reperfusion injury following ischemia [27]. Evidence suggests that sepsis in COVID-19 contributes to hypoxic liver injury, causing a surge in liver biomarkers, which plausibly explains the higher levels of serum ALT, AST, and total bilirubin in severe COVID-19 patients relative to the non-severe group in our study [18,29]. Nonetheless, the involvement of the brainstem by SARS-COV-2 may also affect the respiratory center, causing breathing difficulties and subsequent hypoxemia, further leading to hepatic hypoxia and ischemia in critically ill COVID-19 patients [30]. Hence COVID-19 encompasses an array of problems that could result in liver injury either by directly infecting the liver or causing damage secondary to the involvement of other systems such as the pulmonary, cardiovascular, or neurological system. However, there is still a scarcity of data reporting liver failure in COVID‐19 patients with chronic liver diseases. Future studies are warranted to explore the mechanisms of hepatic dysfunction in patients with COVID-19.

The present meta-analysis, however, has a few limitations. Firstly, most of the studies included in the meta-analysis had a retrospective study design; thus, there is a danger of bias in data collection. Second, since all the studies reported only hospitalized patients, the occurrence of liver injury among COVID-19 patients in our study may have been overestimated. Therefore, our results cannot be extrapolated to the entire population of SARS-CoV-2 patients. Third, many studies reported patients with pre-existing chronic liver disease, which renders them susceptible to developing an acute liver injury. However, the present analysis did not monitor the possible effects of potential confounders, such as age, gender, and comorbidities; hence, the findings must be interpreted with caution. Moreover, the majority of the studies included is from China, and, thus, may not represent variations between different populations.

Despite, the above limitations, this systematic review and meta-analysis provide useful information on the prevalence and liver complications of COVID-19 infection.

## Conclusions

This review comprehensively analyzes the liver enzymes of COVID-19 patients who experienced liver injuries. It also correlates deranged liver biomarkers with the severity of the COVID-19 disease. The analysis revealed that liver function derangements, such as hypoalbuminemia, hyperbilirubinemia, and elevated aminotransferase levels, are common in COVID-19 infection. Moreover, these abnormalities were found to be relatively higher in severe cases of COVID-19 than in non-severe cases. Hence, we conclude that impaired liver biochemistry serves as a prognostic factor to assess COVID-19 severity. Liver markers should, therefore, be observed and monitored continuously to avoid poor outcomes.
